# Assessing tobramycin levels and renal function following the implantation of CaSO_4_ beads impregnated with tobramycin: A prospective cohort study

**DOI:** 10.1097/MD.0000000000032276

**Published:** 2022-12-09

**Authors:** Shuroug A. Alowais, Haytham A. Wali, Deanne Tabb, Ahmad Alamer, Nehad Ahmed, Saeed A. Baloch, John C. P. Floyd

**Affiliations:** a Department of Pharmacy Practice, College of Pharmacy, King Saud bin Abdulaziz University for Health Sciences, Riyadh, Saudi Arabia; b King Abdulaziz Medical City, National Guard Health Affairs, Riyadh, Saudi Arabia; c King Abdullah International Medical Research Center, Riyadh, Saudi Arabia; d Piedmont Columbus Regional Midtown, Columbus, GA; e Department of Pharmacy Practice, College of Clinical Pharmacy, King Faisal University, Al-Ahsa, Saudi Arabia; f Department of Pharmacy Practice, College of Pharmacy, Prince Sattam bin Abdulaziz University, Alkharj, Saudi Arabia; g The Hughston Clinic, Columbus, GA.

**Keywords:** acute kidney injury, calcium sulfate beads, therapeutic drug monitoring, tobramycin

## Abstract

This study aimed to evaluate the risk of serum tobramycin concentrations exceeding therapeutic levels after administration of calcium sulfate (CaSO_4_) beads containing either 240 mg or 400 mg tobramycin and 1000 mg vancomycin. This single-center, prospective. This single-center, prospective study included included Piedmont Columbus, Regional orthopedic surgery patients. Following the implantation of tobramycin into CaSO_4_ beads, serially measured serum tobramycin concentrations were evaluated after 6, 12, 24, and 48 hours. In addition to that, serum tobramycin concentration was evaluated after 5 days. None of the patients who received 240 mg tobramycin-impregnated beads had a tobramycin level >2 μg/mL. Six hours after implantation, the tobramycin level in 2 out of 2 (100%) patients who received 400 mg of tobramycin-impregnated beads was >2 μg/mL. One day following the surgery, the median serum creatinine was 0.85 mg/dL, with an interquartile range of 0.73 to 1.04 mg/dL. No cases of acute kidney injury were observed. This cohort demonstrated that non-nephrotoxic serum tobramycin levels could be achieved in CaSO_4_ beads mixed with 240 mg or 400 mg of tobramycin.

## 1. Introduction

Antibiotic concentrations may be insufficient in poorly vascularized infected areas when taking systemic antibiotics for persistent osteomyelitis or infected fractures. As a result, the standard of care for generating high concentrations in the infected area with less systemic exposure is to use antibiotic-impregnated beads.^[1]^ Calcium sulfate (CaSO_4_) beads have been developed because of the suboptimal antibiotic eluting property of traditional polymethyl methacrylate cement spacers and antibiotic-incorporated beads.^[2]^ Reduced biocompatibility with bone, a short duration of antibiotic release, a very low release rate, heat damage to antibiotics, and the requirement for a second surgical procedure to remove the beads after implantation are further drawbacks of polymethyl methacrylate beads.^[3,4]^ In contrast, CaSO_4_ beads are entirely absorbed at the end of 4 months, removing the possibility of biofilm development on residual beads.^[5]^

After the local application of tobramycin CaSO_4_ beads, systemic exposure was evaluated in a prospective pharmacokinetic study.^[6]^ The study found that tobramycin serum concentrations peaked at 6 hours with an average of 1.5 μg/mL in 12 patients with a mean creatinine clearance of 110 mL/min. At 48 hours, tobramycin levels in patients who received 10 g of CaSO_4_ beads were almost undetectable. However, it took 120 hours for patients who received 20 g of CaSO_4_ beads containing 800 mg of tobramycin to disappear. One patient in the 400 mg group with a creatinine clearance of 10 mL/min was at risk of persistently elevated tobramycin levels. Tobramycin levels could remain >2 μg/mL for estimations of creatinine clearance of <30 mL/min, according to simulation research that highlighted the potential toxicity of up to 2000 mg of the drug.^[6]^

Tobramycin trough levels >2 μg/mL that are persistently elevated are undesirable as they may be linked to an increased risk of renal impairment. Gentamicin levels of >2 μg/mL were demonstrated to be an independent predictor of gentamicin-associated acute kidney damage in a previous retrospective observational study of patients who were treated with the antibiotic (odds ratio 1.845, 95% confidence interval 1.22–2.79).^[7]^ Tobramycin has been shown to have less nephrotoxicity than gentamicin,^[8,9]^ but nephrotoxicity has been recorded in 22% of individuals with an average tobramycin trough concentration of 2.2 μg/mL.^[10]^

A case report of acute tubular necrosis with a high tobramycin level following the implantation of CaSO_4_ beads containing 2.4 g of tobramycin and 1 g of vancomycin was reported at the beginning of 2019. Prior to surgery, the patient’s baseline serum creatinine level was 1.13 mg/dL, and his vancomycin trough level was 13.4 μg/mL. After bead implantation, the patient’s serum creatinine rose to 5.21 mg/dL. On the first and second postoperative days (POD), the vancomycin levels were 40.9 and 37.4 μg/mL, respectively, while the tobramycin level was 15.9 on POD2. After surgery, the serum creatinine level increased to 8.28 mg/dL on POD5, and the tobramycin level decreased to 5 μg/mL. Over time, renal function without dialysis improved.^[11]^

It is interesting to note that the company that produces Stimulan® beads does not support the addition of antibiotics to its bone filler product. However, tobramycin is listed in 2 doses on the product insert: 240 mg for the reconstituted intravenous solution and 1.2 g for the powder formulation.^[12]^ According to the manufacturer, mixing these 2 tobramycin concentrations into the final compounded CaSO_4_ bead product does not affect its consistency. The current study characterized the serum concentrations of tobramycin and examined changes in renal function following implantation of CaSO_4_ beads mixed with 240 mg tobramycin and 1000 mg of vancomycin.

## 2. Methods

This single-center prospective study assessed the serum concentration of tobramycin after implantation of tobramycin-impregnated CaSO_4_ beads from patients undergoing orthopedic surgery at Piedmont Columbus Regional Midtown in Columbus, Georgia, from November 2019 to September 2021. When implanting tobramycin-impregnated CaSO_4_ beads during orthopedic surgery, adult patients (>18 years of age) were included. Patients who received intravenous tobramycin postoperatively were excluded.

The infectious disease pharmacy service was informed of the orthopedic surgeons’ recruitment of suitable patients for tobramycin-level monitoring. Beads were prepared in the operation room during surgery, typically using a 10-mL kit containing 20 g of a pharmaceutical-grade CaSO_4_ alpha-hemihydrate powder (Stimulan®, Biocomposites Ltd., Keele, UK). The beads were mixed with 1000 mg of vancomycin hydrochloride powder and 6 mL of a 40 mg/mL (240 mg) tobramycin solution. The final amount of antibiotics administered to each patient was communicated to the infectious disease pharmacist by the operating room nurse or obtained from the chart after surgical completion. Following patient consent, serial tobramycin levels were obtained at 6, 12, 24, 48 hours, and 5 days after implantation. The remaining levels were canceled if any level was <1 μg/mL. Daily serum creatinine, starting from the admission date until the last tobramycin level is obtained, was used to monitor kidney function. Although serum creatinine is a delayed biomarker for kidney injury, the availability of more accurate biomarkers at the hospital and the turnaround time to get the results affected their feasibility. The primary endpoint was the percentage of patients with persistently elevated tobramycin levels >2 μg/mL after 48 hours. The secondary objectives were the incidence of acute kidney injury and its severity when it occurred in the presence of consistently elevated tobramycin levels according to the “Kidney Disease: Improving Global Outcomes” criteria.^[13]^

The Piedmont Healthcare Institutional Review Board approved this study. Informed consent was obtained from all study participants before the levels were ordered. Descriptive statistics were used to represent the study’s findings. Categorical data are presented as frequencies and percentages, while continuous data are presented as a means with standard deviations (SDs) or medians with interquartile ranges.

## 3. Results

### 3.1. Baseline characteristics of the study participants

During the study period, we identified 23 patients. Of these, 19 met inclusion criteria. One patient was excluded because of age <18 years, and 3 were excluded because of an inability to consent. The baseline characteristics of the patients are presented in Table 1. The average age was 43.9 years (SD, 17.2 years). Males accounted for 63.2% of the participants. The average baseline serum creatinine was 0.9 mg/dL (SD, 0.19 mg/dL). Ninety percent (17/19) of patients received 10 mL (20 g) of CaSO_4_ beads mixed with 240 mg tobramycin and 1000 mg of vancomycin. The remaining 2 patients received 20 mL (40 g) of CaSO_4_ beads mixed with 400 mg tobramycin and 1000 mg vancomycin.

**Table 1:** Baseline characteristics of the study participants.

Characteristic	Cohort (N = 19)
Age (yr), mean (SD)	43.9 (17.2)
Male, n (%)	12 (63.2)
Weight (kg), mean (SD)	97.2 (35.7)
Body mass index, n (%)	
<30 kg/m^2^	9 (47.4)
>30 kg/m^2^	10 (52.6)
Baseline serum creatinine (mg/dL), mean (SD)	0.9 (0.19)
Race, n (%)	
White	11 (57.9)
African American	8 (42.1)
Nephrotoxic drugs prior to the procedure, n (%)	
Vancomycin	8 (42.1)
Gentamicin	1 (5.3)
IV contrast dye	1 (5.3)
None	9 (47.4)
Sites of implantation, n (%)	
Ankle	2 (10.5)
Arm	1 (5.3)
Elbow	1 (5.3)
Foot	2 (10.5)
Hip	2 (10.5)
Knee	2 (10.5)
Leg	1 (5.3)
Spine	4 (21)
Tibia	3 (15.8)
Ulna	1 (5.3)
Tobramycin amount in CaSO_4_ beads, n (%)	
400 mg*	2 (10.5)
240 mg	17 (89.5)

CaSO_4_ = calcium sulfate, IV = Intravenous, SD = standard deviation.

* Mixed with an amount of 20 mL (40 g) of CaSO4 beads.

### 3.2. Tobramycin serum levels after CaSO_4_ beads implantation

We evaluated the serum levels of tobramycin post bead placement to determine if any were >2 μg/mL at specified time points. Two patients had a tobramycin level >2 μg/mL 6 hours after implantation (2.5 and 2.7 μg/mL, respectively). In both cases, the tobramycin dose was 400 mg. The serum creatinine on POD1 was 0.89 and 0.66 mg/dL in the first and second cases, respectively. In the first case, the tobramycin level decreased to <1 μg/mL after 12 hours. Therefore, no other levels were obtained according to the study protocol. In the second case, tobramycin levels were sustained at >2 μg/mL at 24 hours. The level declined from 2.7 μg/mL down to 2.4 μg/mL within 18 hours. Unfortunately, the patient was discharged before additional levels were obtained (Table 2). In light of this patient’s normal kidney function and rate of tobramycin decline, it was anticipated that the tobramycin level would be 2 μg/mL or less by 48 hours. Therefore, none of the patients in this cohort met the primary endpoint of sustained elevated tobramycin levels >2 μg/mL after 48 hours. Moreover, no cases of acute renal injury were observed.

**Table 2:** Tobramycin serum levels after calcium sulfate beads implantation.

Patient	Tobramycin serum concentration (μg/mL)				
	6 h	12 h	24 h	48 h	5 d
1	–	1	<0.3	–	–
2	2.7	–	2.4	–	–
3	2.5	0.5	–	–	–
4	0.5	<0.3	–	–	–
5	<0.3	<0.3	–	–	–
6	0.5	<0.3	–	–	–
7	<0.3	–	<0.3	–	–
8	<0.3	<0.3	–	–	–
9	<0.3	<0.3	–	–	–
10	0.6	<0.3	–	–	–
11	1	0.7	0.3	–	–
12	<0.3	–	<0.3	–	–
13	<0.3	–	<0.3	–	–
14	–	0.8	–	–	–
15	<0.3	<0.3	–	–	–
16	0.5	–	–	–	–
17	0.7	<0.3	–	–	–
18	0.5	<0.3	–	–	–
19	<0.3	<0.3	–	–	–

### 3.3. Serum creatinine levels of participants during admission

The serum creatinine levels before and after surgery of the included patients are presented in Table 3. One patient had an acute elevation in serum creatinine before the procedure (4.92 mg/dL). The patient had no history of chronic kidney disease. Despite the undergoing procedure and the tobramycin impregnated CaSO_4_ beads placement, the serum creatinine was trending down postoperatively (4.57 and 4.39 mg/dL on POD1 and POD2, respectively), and the serum tobramycin levels were <0.3 μg/mL on both days.

**Table 3:** Serum creatinine levels of participants during admission.

Patient	Baseline SCr (mg/dL)	SCr prior to procedure (mg/dL)	SCr POD1 (mg/dL)	SCr POD2 (mg/dL)
1	1.09	0.99	1	1.19
2	–	–	0.89	–
3	0.66	0.52	0.66	0.63
4	0.67	0.7	0.61	0.66
5	0.62	0.68	–	–
6	1.11	0.95	1.05	0.88
7	1.26	1.22	1.33	1.26
8	0.72	0.51	0.73	–
9	1.07	0.93	1.29	0.94
10	0.78	0.78	0.71	0.54
11	0.85	0.85	0.67	0.65
12	0.97	1.19	1.05	–
13	1.07	1.16	0.83	0.84
14	0.7	0.7	0.76	0.72
15	–	4.92	4.57	4.39
16	0.89	0.89	0.77	–
17	1.2	1.2	0.98	–
18	0.91	0.91	0.86	–
19	0.71	0.65	0.73	–

POD = postoperative day, SCr = serum creatinine.

## 4. Discussion

To our knowledge, this is the first study to assess the systemic levels of tobramycin and the renal safety of using tobramycin loaded CaSO_4_ beads. Our study demonstrated tobramycin absorption into the systemic circulation in more than half of the cases (57.8% [11/19]). While most of the levels were <1 μg/mL, 2 cases had peak levels >2 μg/mL. None of the patients experienced a significant increase in serum creatinine level following implantation. Our findings on tobramycin levels are consistent with a previous study that assessed the elution profile of tobramycin and vancomycin in CaSO_4_ beads.^[14]^ Although our study did not evaluate serum vancomycin levels after placing the beads, the absence of serum creatinine elevation after implantation could indicate that vancomycin did not reach nephrotoxic levels.

Based on the Stimulan® Rapid Cure Powder package insert, commonly used doses of tobramycin include 1.2 g in powder formulation and 240 mg when used as a reconstituted solution.^[12]^ Additionally, the insert describes that when combined with vancomycin 1-g powder, the dose of tobramycin is 240 mg of liquid. Stimulan® kits were available in 3 sizes: 5 mL, 10 mL, and 20 mL. The most commonly used size is the 10-mL kit, and the antibiotic doses are based on that size. There is no mention of alternative dosing when using different kits. In our study, 3 patients required 20 mL of CaSO_4_ powder. Two patients received 400 mg of tobramycin, and the 6-hour peak was >2 μg/mL in both cases. Even though the 6-hour tobramycin level for the patients who received 400 mg of tobramycin was >2 μg/mL, neither patient had an elevated serum creatinine on POD1. Concerns for elevated tobramycin levels should be considered in patients receiving doses >240 mg and in those with preexisting renal dysfunction or risk factors for renal insufficiency.

The Stimulan® manufacturer does not endorse the use of antibiotics added to their bone filler and further states that it is considered an off-label use. The goal of including antibiotic doses in the package insert was to inform how the antibiotic dose affects the bead set times. Furthermore, the elution and absorption of antibiotics from CaSO_4_ beads can be affected by the amount of antibiotic added, underlying renal dysfunction, and vascularity at the implantation site. Additionally, the combination of antibiotics could affect the elution properties of each antibiotic. Specifically, tobramycin was found to extend the elution of vancomycin from the CaSO_4_ beads to 40 versus 20 days when vancomycin was added alone to the CaSO_4_ beads.^[15]^

The median serum creatinine level 1 day after the procedure, was 0.83 mg/dL (interquartile range, 0.73–1.05 mg/dL) (Table 3). When assessing the maximum level of tobramycin for each patient, we found that the maximum level of tobramycin occurred in most patients 6 hours post-surgery (Table 2). A previous in vitro elution study using 3 g of CaSO_4_ beads immersed in phosphate-buffered saline found that tobramycin had eluted to the peak concentration at the 4-hour time point.^[16]^ Another study used 2 g of CaSO_4_ beads in a Dulbecco Modified Eagle medium, a physiological solution that closely mimics the in vivo environment, and found that the peak concentration of tobramycin was achieved at an 8-hour time point.^[17]^ The peak level in our study was reached within the time range (4–8 hours) reported in previous studies. The tobramycin level at 6 hours post-surgery ranged from undetectable (<0.3 μg/mL) up to 2.7 μg/mL.

Our study did not assess the efficacy of a 240 mg dose of tobramycin in the CaSO_4_ beads. However, a previous study showed that using a 240 mg dose of tobramycin resulted in an average local concentration of 31 μg/mL on POD1 and 4.6 μg/mL on POD5, which is well above the minimum inhibitory concentration of the gram-negative organisms targeted by tobramycin.^[14]^ Another study that used the same amount of vancomycin and tobramycin in CaSO_4_ beads used in our study found significantly reduced methicillin-resistant *Staphylococcus aureus* surface colonization, with an initial 3-log reduction in the number of CFU/cm^2^ at day 1 (24 hours post-inoculation).^[18]^ These previous findings indicate that the antibiotics concentrations used in the current study are effective despite the absence of a direct measure.

This study has several limitations. First, the sample size was small. However, the recruitment for this study was done during the coronavirus disease 2019 pandemic. The elective surgeries at the hospital were postponed then, resulting in low recruitment. Nevertheless, we believe that the findings from the small sample of our study can help generate ideas for further research in this field. Second, the implantation of CaSO_4_ beads may be complicated by seroma formation and wound leakage; however, our study could not evaluate this complication. Third, the timing for obtaining tobramycin levels was inconsistent. Fourth, we had 1 patient with a 24-hour elevated tobramycin level who was discharged before the 48-hour tobramycin level could be collected. Fifth, our data represent the pharmacokinetics of tobramycin from a specific commercial product in use, with a particular synthesis and elusion rate that might not apply to other commercial products. Sixth, the impact of vancomycin absorption from bead placement could not be assessed, because vancomycin levels were not obtained as part of the study.

## 5. Conclusions

Our study results showed that using a tobramycin dose of 240 mg with CaSO_4_ beads in patients undergoing orthopedic procedures resulted in non-nephrotoxic serum tobramycin levels. Most of the patients’ serum tobramycin levels were undetectable 12 hours after surgery. None of the participants developed acute kidney injury during the immediate follow-up period.

## Acknowledgment

The authors would like to thank the chemistry laboratory staff at Piedmont Columbus Regional Midtown for their cooperation in incorporating the tobramycin trough level processes into their workflow for the purpose of our study.

## Author contributions

**Conceptualization:** Shuroug A. Alowais, Deanne Tabb, John C. P. Floyd.

**Data curation:** Shuroug A. Alowais, Haytham A. Wali, Deanne Tabb, Saeed A. Baloch, John C. P. Floyd.

**Formal analysis:** Shuroug A. Alowais, Haytham A. Wali.

**Investigation:** Shuroug A. Alowais, Haytham A. Wali, Deanne Tabb, Saeed A. Baloch, John C. P. Floyd.

**Methodology:** Shuroug A. Alowais, Deanne Tabb.

**Project administration:** Shuroug A. Alowais, Haytham A. Wali, Deanne Tabb.

**Resources:** Saeed A. Baloch, John C. P. Floyd.

**Supervision:** Shuroug A. Alowais, Haytham A. Wali, Deanne Tabb, John C. P. Floyd.

**Validation:** Shuroug A. Alowais, Haytham A. Wali.

**Visualization:** Shuroug A. Alowais, Haytham A. Wali, Deanne Tabb, Saeed A. Baloch, John C. P. Floyd.

**Writing – original draft:** Shuroug A. Alowais, Haytham A. Wali.

**Writing – review & editing:** Shuroug A. Alowais, Haytham A. Wali, Deanne Tabb, Ahmad Alamer, Nehad Ahmed, Saeed A. Baloch, John C. P. Floyd.

All authors have read and agreed to the published version of the manuscript.
